# A perspective on the evidence for glymphatic obstruction in spaceflight associated neuro-ocular syndrome and fatigue

**DOI:** 10.1038/s41526-024-00365-9

**Published:** 2024-02-28

**Authors:** Grant Alexander Bateman, Alexander Robert Bateman

**Affiliations:** 1 Department of Medical Imaging, John Hunter Hospital, Newcastle, NSW Australia; 2 Newcastle University Faculty of Health, Callaghan Campus, Newcastle, NSW Australia; 3 School of Mechanical Engineering, University of New South Wales, Sydney, NSW Australia

**Keywords:** Physiology, Neuroscience

## Abstract

Spaceflight associated neuro-ocular syndrome (SANS) alters the vision of astronauts during long-duration spaceflights. Previously, the current authors have discussed the similarities and differences between SANS and idiopathic intracranial hypertension to try to elucidate a possible pathophysiology. Recently, a theory has been advanced that SANS may occur secondary to failure of the glymphatic system caused by venous dilatation within the brain and optic nerves. There is recent evidence to suggest glymphatic obstruction occurs in childhood hydrocephalus, multiple sclerosis and syringomyelia due to venous outflow dilatation similar to that proposed in SANS. The purpose of the current paper is to discuss the similarities and differences between the known CSF and venous pathophysiology in SANS with these other terrestrial diseases, to see if they can shed any further light on the underlying cause of this microgravity-induced disease.

## Introduction

Spaceflight-associated neuro-ocular syndrome (SANS) refers to the pathological effects of long-term microgravity on the eyes and orbital physiology of astronauts. The clinical manifestations of SANS include unilateral and bilateral optic disc edema, globe flattening, choroidal and retinal folds, hyperoptic refractive error shifts, and focal areas of ischemic retina^1^. In a previous paper, it was discussed whether SANS may be similar to the terrestrial disease idiopathic intracranial hypertension (IIH)^2^. IIH is characterized by an increased intracranial pressure (ICP) in the absence of parenchymal brain lesions, vascular malformations, hydrocephalus or CNS infection^3^. To diagnose IIH, a CSF pressure above 25 cmH_2_O is required^4^. Similar to IIH, long-duration astronauts show evidence of globe flattening, optic nerve protrusion (optic disc edema) and pituitary flattening^5^. A hallmark of IIH is recurrent headaches and although it has been claimed astronauts do not complain of headaches^6^, there are reports of an increase in headaches associated with microgravity, especially during the initial period in space^7^. Three long-duration astronauts underwent post flight lumbar puncture revealing pressures of 23, 28 and 29 cm H_2_O^5^, with 2 of these pressures being in the diagnostic range for IIH. However, there are some significant discrepancies between IIH and SANS. Astronauts do not complain of pulsatile tinnitus unlike IIH patients^6^. IIH presents more often with bilateral eye changes, predominately in women, but SANS has a higher asymmetric or unilateral presentation and is more common in men^1^. Transient visual obscurations or diplopia secondary to a nonlocalising sixth nerve palsy have never been reported in astronauts with SANS, unlike in IIH^1^. The latter suggests there is likely to be more to discover about the pathophysiology of SANS.

**Fig. 1:**
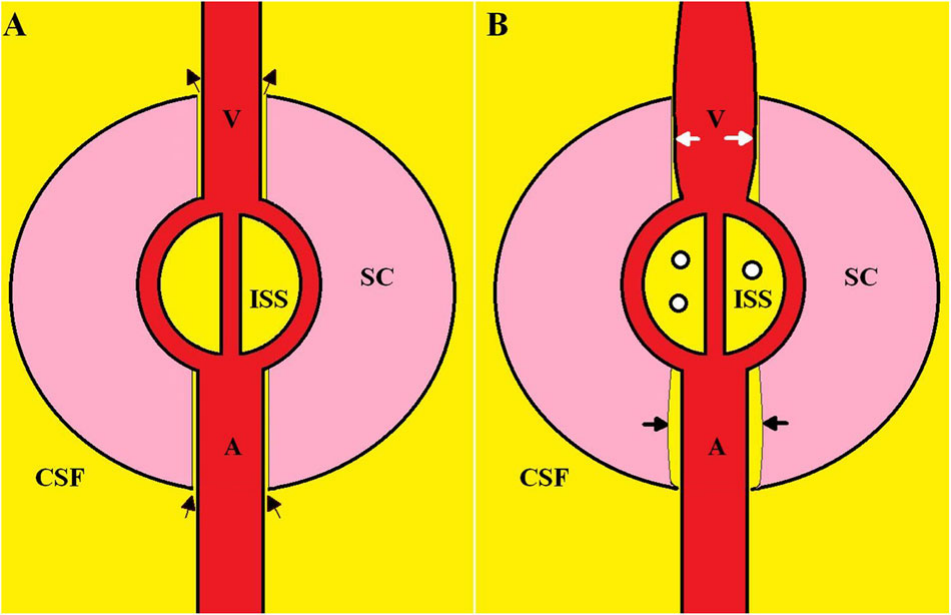
A simplified diagrammatic representation of the glymphatic system with changes occurring in syringomyelia. Panel **A** is the normal physiology. SC is the spinal cord, CSF is the surrounding cerebrospinal fluid, A is the arteriolar inflow, V is the vein draining the spinal cord and ISS is the interstitial space. CSF enters the space between the artery and spinal cord via the spinal cord arterial perivascular space (lower black arrows), passes through the interstitial space and exits via the venous perivascular space (upper black arrows). Panel **B** shows the changes associated with syringomyelia. There is a dilatation of the vein compressing its perivenular space (white arrows). There is build-up of interstitial fluid increasing the ISS pressure and there is coalescence of small cystic spaces indicating the developing syrinx (small circles). The back-up of interstitial fluid dilates the spinal cord arteriolar perivascular spaces (black arrows). Reproduced from Bateman and Bateman^15^ under a Creative Commons Attribution (CC BY) license.

Recently, Wostyn et al. have hypothesised that dilatation of the cerebral perivascular spaces (PVS) in the brains of long-duration space travellers may result from altered hemodynamics leading to obstruction of the perivenous glymphatic outflow^8^. An increase in the size of the MRI visible cerebral perivascular spaces is thought to reflect impaired glymphatic exchange^9^. There is evidence of increased PVS size in first-time astronauts postflight^10^, as well as increased PVS size in those who develop SANS^11^. The glymphatic system is thought to support a continuous movement of fluid from the subarachnoid space through the brain or spinal cord parenchyma and back into the subarachnoid space via the perivenous space^12^. In the glymphatic system, CSF enters the periarterial vascular space (which is limited by the pia mater) via arterial pulse-induced convection. From here, it enters the interstitial space through gaps in the astrocyte end-foot processes and via aquaporin 4 water channels. Water exits the interstitial space via the perivenous space^12^ (see Fig. 1A). The perivenous drainage of interstitial solutes provides these solutes access to the sinus-associated lymphatics, either directly since these large veins merge to form the dural sinuses, or indirectly via the cisternal CSF compartments associated with these structures. In this sense, it may be appropriate to regard these two components, perivascular pathways within the CNS parenchyma and the extra-axial meningeal lymphatic vessels as serial elements of a wider functional system^13^. The current authors have suggested that there is likely to be obstruction of the glymphatic system, due to dilatation of the venous outflow. This dilatation has been shown to occur in childhood hydrocephalus, multiple sclerosis and syringomyelia and obstruction occurs because of a shared outflow geometry^14,15^ (see Fig. 1B). This is similar to the hypothesis suggested by Wostyn et al. for SANS^8^. The purpose of the current paper is to compare and contrast the CSF and venous outflow findings in SANS with these latter three terrestrial diseases to see if there is any information to be gleaned.

## PHYSIOLOGY OF SANS

As discussed above, there are similarities and differences in the manifestations of IIH and SANS. The main hypothesis regarding the cause of SANS centres on a rise in ICP due to the cephalad fluid shifts occurring during long-duration spaceflight^1^. A second hypothesis suggests there is compartmentalisation of the CSF within the optic nerve sheath with a locally increased CSF pressure^1^. The latter would not explain the elevated CSF pressures at lumbar puncture found in long-term space flight. It was hypothesised that an increase in ICP in spaceflight could be due to a combination of an elevation in jugular vein pressure from these cephalad fluid shifts^1^, together with an increase in venous pressure secondary to an increase blood flow through the cerebral venous system, occurring due to an elevation in the inspired CO_2_ levels in flight^2^. A well-recognised terrestrial microgravity analogue is head-down tilt, which simulates the cephalad fluid shifts. In a recent head-down tilt study, there was an increase in ventricular and perivascular space volume suggesting glymphatic dysfunction but decreased cerebral blood flow^16^. Despite the findings of decreased blood flow in head-down tilt experiments, there is evidence for an increase in the cerebral blood flow during spaceflight in the literature. We are unable to suggest a reason for this discrepancy in the cerebral blood flow between head-down tilt and spaceflight. However, there is a 41-56% increase in cardiac output^17,18^, an 83% increase in straight sinus blood flow velocity^19^ and an 84% increase in the middle cerebral vein flow velocity in long-term space flight^20^. More recently, chronic hemolytic anemia and a reduction in haemoglobin concentration of about 10% have been noted in long term spaceflight^21^. Whilst it has long been thought that IIH is associated with either a normal or reduced cerebral blood flow (CBF), it has also recently been shown that there are a subset of patients with IIH who have an increased CBF i.e. cerebral hyperemia secondary to the anemia^22^. Approximately 20% of IIH patients are anemic and this is a potent cause of increased blood flow^22^. The CSF volume, venous volume and glymphatic findings in the diseases to be discussed are summarised in Table 1. Similar to SANS, in IIH there is dilatation of the PVSs suggesting glymphatic dysfunction^23^. However, in addition to the differing clinical findings between IIH and SANS as already discussed, there are also differences in the pathophysiology. In IIH there is a normal brain ventricular volume but the extra-ventricular CSF volume is significantly increased^24^. This compares to a study of prolonged spaceflight, where the opposite was found i.e. evidence of dilated lateral ventricles and a decreased subarachnoid space at the vertex^11^. The latter CSF volume changes correlated with the enlarged white matter cerebral perivascular spaces^11^ and therefore with the glymphatic dysfunction. The authors of the paper speculated the reduction in vertex CSF volume could obstruct the CSF and interstitial fluid efflux routes (arachnoid granulations, superior sagittal sinus and bridging veins)^11^. However, there is evidence of an increase in the sinus size in SANS and not a decrease^25^. In spaceflight, there is an increase in ventricular size ranging from 11-25% and this enlargement persists for up to three years postflight^26^.

**Table 1:** Summary of findings

	Idiopathic Intracranial Hypertension	Hydrocephalus	Multiple Sclerosis	Syringomyelia	Spaceflight Associated Neuro-optic Syndrome
Ventricle volume	Normal	Enlarged	Enlarged due to atrophy	Dilatation of the syrinx	Enlarged
Subarachnoid space volume	Enlarged	Reduced	Unknown probably enlarged due to atrophy	Reduction in SAS around cord	Reduced
Sinus/ cortical venous size	Sinus normal or reduced, cortical veins unknown	Reduced Sinus enlarged cortical veins	Enlarged Sinus and cortical veins	Enlarged subarachnoid veins	Enlarged sinus, possibly cortical veins
Glymphatics	Obstructed	Obstructed	Obstructed	Obstructed	Obstructed

## Similarities between SANS and hydrocephalus

**Fig. 2:**
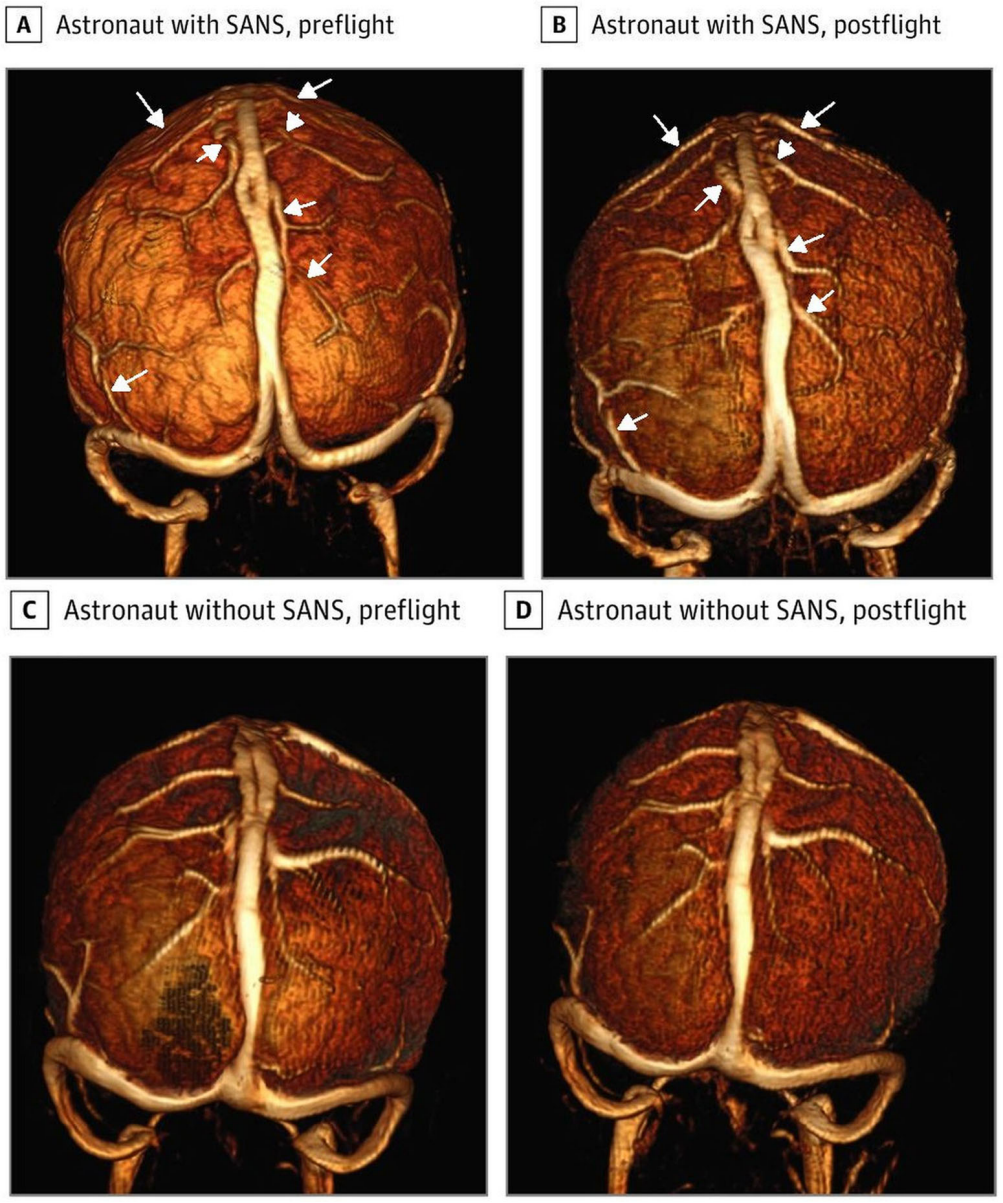
Venous dilatation in SANS. Three-Dimensional reconstructions of the preflight and postflight MR venograms for an astronaut with spaceflight-associated neuro-ocular syndrome (SANS) and an astronaut without SANS. There is some dilatation of the venous sinuses in the astronaut with SANS postflight (**A**, **B**) but none seen in the astronaut without SANS (**C**, **D**). The arrows highlight the cortical venous segments which are larger post-flight in the SANS affected astronaut compared to pre-flight (**A**, **B**). Reproduced from Rosenberg et al.^25^ under a Creative Commons Attribution (CC BY) license.

The dilatation of the ventricles and narrowing of the subarachnoid space over the vertex, as found in long-duration spaceflight, are more in keeping with communicating hydrocephalus^27^ than IIH. Similar to SANS, in childhood hydrocephalus, 13% of individuals have cerebral hyperemia^28^. Hydrocephalus has also been noted to reduce the glymphatic flow^29^ similar to SANS. Thus, SANS may have more in common with hydrocephalus than IIH. In childhood hydrocephalus, the bridging cortical veins are found to be 22% larger than in controls^28^. Dilatation of the outflow veins of the brain has been suggested to obstruct the glympathic pathway because they share a common outflow geometry^15^ (see Fig. 1B). This is similar to the hypothesis put forward by Wostyn et al.^8^. However, there are also differing venous physiological findings between SANS and hydrocephalus. In a study comparing the magnetic resonance venograms in astronauts both pre-flight to 2 days postflight (the flight time averaging 184 days), those with SANS showed a 13.4% increase in the superior sagittal sinus volume, 17.15% increase in right transverse/ sigmoid sinus volume and 9.4% increase in left transverse sinus/ sigmoid volume (see Fig. 2). In the non-SANS individual’s, the changes in volume were -2.66%, 0.77% and -1.4%, respectively^25^. This difference in size between the groups is significant and is likely at the heart of the pathophysiology of SANS. This contrasts with childhood hydrocephalus, where there is the opposite finding i.e. a reduction in the cross-sectional area of the sagittal sinus of 35%, a 30% reduction in the transverse sinuses and 41% reduction in the sigmoid sinuses compared to controls^28^. An increase in the CSF outflow resistance in hydrocephalus will increase the CSF pressure with respect to the venous pressure^30^ (i.e. the transmural pressure) accounting for the sinus compression in hydrocephalus. Thus, SANS is associated with an increase in the size of the sinuses but hydrocephalus is associated with a decrease in them. However, as a counterpoint to this finding, we note in the figure from Rosenberg et al.’s paper where SANS sinus dilatation was discussed^25^, there are hints at a possible dilatation of the cortical veins in SANS similar to childhood hydrocephalus (see Fig. 2A, B). It can be seen that in the astronaut destined to develop SANS, the pre-flight sagittal, transverse sinuses and cortical veins were smaller than in the postflight image, but no change can be seen in the non-SANS individual. The cortical veins were not discussed in this paper; however, they appear enlarged in the postflight SANS individual. This suggests the data may already exist to answer the question as to whether the cortical veins are dilated in SANS or not. The cause of the dilatation of the venous sinuses in SANS is difficult to reconcile. The authors suggested there may be venous obstruction downstream, outside of the cranium or venous laxity in the SANS individuals, in comparison to the non-SANS individuals, to account for the findings but also noted the difficulty in explaining the dilatation given that most astronauts appear to have an elevated post-flight ICP^25^. The CSF pressure should always stay above the venous sinus pressure for CSF to drain via this route^30^. The free walls of the sinuses are fixed at their inner attachments to the falx or tentorium cerebri and also their outer attachments to the inner aspect of the skull^31^. The sinus-free walls are viscoelastic structures and collapse inward or dilate outward with the degree of movement, depending on the pressure across their walls (the transmural pressure), their intrinsic wall stiffness and the wall thickness^31^. In hydrocephalus where the sinus transmural pressure is increased secondary to the elevated resistance to CSF absorption across the sinus wall^32^, the cross-sectional area of the sagittal sinus is decreased by 25%^33^. In idiopathic intracranial hypotension where the CSF pressure is significantly decreased below the normal level of 11.4 mmHg to 4.4 mmHg^34^ (and also below the normal venous sinus pressure of 7.5 mmHg^33^), the area of the sinus is increased by 22%^33^. Note the CSF pressure can only be below the venous sinus pressure in idiopathic intracranial hypotension due to another CSF outflow pathway (i.e. a leak). As already discussed, the ICP is likely to be elevated in long-flight astronauts when evaluated post flight but the cephalad fluid shifts have likely already reversed by then. In flight, the jugular vein pressures are high^35^ but postflight, the jugular vein volumes rapidly return to those as seen before the flight^36^. The walls of the sinuses are passive structures and should react instantly to any change in their transmural pressure. A probable increase in the transmural pressure postflight (high ICP but normal jugular vein pressure) should reduce the size of the sinuses and not increase them (as seen in hydrocephalus). Further, any increase in the sinus wall compliance (reduction in stiffness) as suggested by Rosenberg et al. would make the sinuses even smaller. To highlight this discrepancy, in IIH the sagittal sinus transmural pressure was found to be low-normal at 2.34 mmHg in one paper^37^ and 2.7 mmHg in another^38^ (normal is 4 ± 2 mmHg^39^) and because of this, the size of the sagittal sinus is also normal in IIH^33^. The sinuses further downstream (transverse sinuses) in IIH are often collapsed because 1) the veins appear to be more compliant at the distal collapse site and 2) the transmural pressure increases as one passes along the sinuses because the sinus pressure must be reduced with distance to maintain the blood flow, but the ICP is the same everywhere^40^. Dilated sinuses do occur in intracranial hypotension where the transmural pressure is reversed as discussed^33^ but a low ICP does not appear to exist in SANS. The sinus size discrepancy indicates there is yet more to the causation of SANS than just an elevated jugular vein or sinus pressure.

## Similarities between SANS and multiple sclerosis

The ventricle size in MS is increased over time but this is due to atrophy^41^, the subarachnoid space volume has not been measured but is likely also increased due to atrophy. Unlike IIH and hydrocephalus, the sinuses are larger in multiple sclerosis than in controls^33^ similar to SANS. An obvious discrepancy with SANS is the autoimmune reaction in MS, but we are mostly interested in the venous manifestations. Vascular modelling in MS has shown that an increase in the pressure within the neck veins or major sinuses is minimal but there is evidence of a slight elevation in ICP^31^. Thus, similar to SANS individuals whilst back on the ground, in MS the transmural pressure is normal or slightly increased but the sinuses dilate anyway. This finding demands an engineering solution. The only way to reconcile this is to hypothesise the sinus walls are either stiffer than normal and/or their walls are thicker than normal^31^. The same explanation would be required to explain the increased sinus size in SANS. However, no direct measurements of the wall thickness or stiffness in MS or SANS have been attempted. Of interest, MS shares some common CSF and hemodynamic physiology with hydrocephalus^42^ suggesting the commonality of the findings between SANS and hydrocephalus may be retained. We are unable to speculate why the sinus walls would be stiffer or thicker than normal in SANS.

There is evidence of glymphatic outflow disruption in MS^43,44^ and also evidence of cortical venous outflow dilatation. In multiple sclerosis, the superficial cortical veins were 29% larger and the vein of Galen 25% larger in cross-sectional area than in the controls^45^. Despite the cortical vein dilatation, no evidence of obstruction at the outflow to the sagittal sinus or straight sinus was seen^45^. Modelling of the cortical vein dilatation found could only be due to an increase in transmural pressure (a reduction in wall thickness or stiffness were not feasible). The normal internal pressure within the cortical veins is higher than the ICP (the opposite of the sinus wall transmural pressure direction), indicating the cortical vein pressure in MS and SANS must be much higher than the elevated ICP for them to dilate. An impedance pump model was suggested to account for the finding of increased pressure localised to the cortical vein segments, which cannot be accounted for by the ICP or sinus pressure^45^. Thus, in SANS there is likely to be structural changes in the sinus walls, altering their impedance. Similar to MS, an increase in sinus wall stiffness would be expected to alter the impedance matching between the cortical veins and their outflow pathway into the sinuses, increasing their pressure and dilating them. Venous outflow dilatation has been correlated with glymphatic obstruction in MS^14^. However, although the above findings can explain the brain findings in SANS, it would be expected that a global reduction in intracranial compliance from an elevation in ICP and sinus wall thickening/ stiffening should affect both optic nerves equally. However, as described previously, the findings in SANS are often unilateral or asymmetrical^1^.

## Similarities between SANS and syringomyelia

**Fig. 3:**
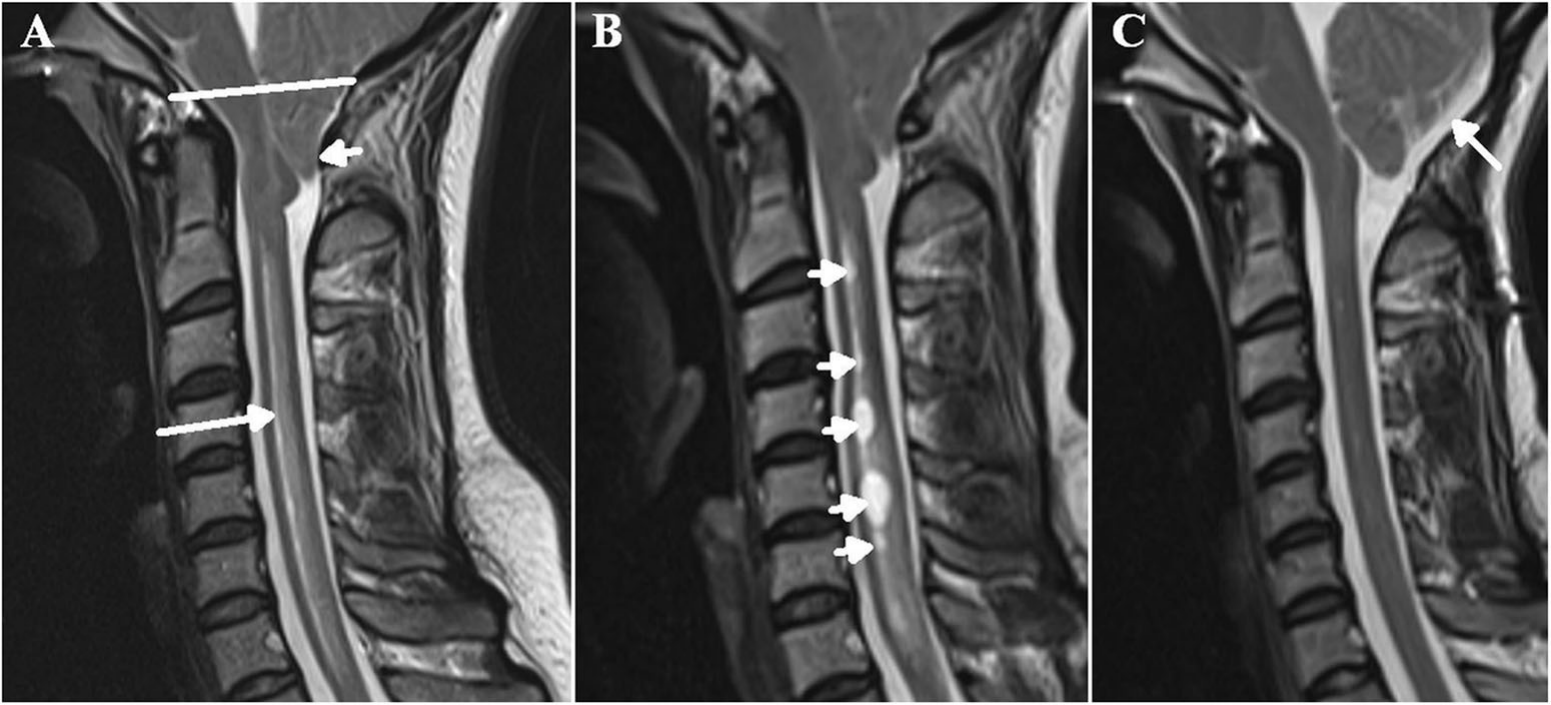
The progression of edema in syringomyelia. A T2 weighted sagittal MRI of the neck in a 28-year-old female patient who presented with headache on bending and coughing. There is a Chiari 1 malformation with significant cerebellar tonsil herniation (small white arrow) below the foramen magnum (white line) leading to reduced spinal canal compliance. There is spinal cord edema from C2 to T1 (long arrow) (**A**). One year later, following conservative management, there were multiple cystic cavities developing (arrows) (**B**). Following posterior fossa decompression (arrow), there is now improved compliance in the spinal canal with the resolution of the edema and the cystic cavities previously noted (**C**). Reproduced from Bateman and Bateman^15^ under a Creative Commons Attribution (CC BY) license.

Syringomyelia is the development of a tubular, fluid-filled cavity within the parenchyma of the spinal cord^46^. The dilated cavity is analogous to the ventricular dilatation as seen in SANS and leads to a reduction in the subarachnoid space around the cord at that level^47^ also similar to SANS. Syringomyelia is commonly associated with an intradural, extramedullary obstruction such as the tonsillar herniation found in Chiari 1 malformation^46^. Thus, in syringomyelia there is isolation of the spinal canal and reduced compliance of this isolated section (see Fig. 3A). In Chiari 1 malformation with syrinx formation, the local cervical compliance is reduced by 45% with a 44% increase in CSF pulse pressure^48^. Many animal models of syringomyelia indicate probable glymphatic malfunction^15^ and there is a known association between syringomyelia an MS^49^. There is evidence of venous dilatation within the subarachnoid space around the cord in MS^50^. In a kaolin-induced dog model of syringomyelia, microangiograms show evidence of venous engorgement surrounding the cord^51^. Thus, isolation of the spinal canal and a reduced compliance are associated with dilation of the draining veins and a reduction of the glymphatic flow, similar to that hypothesised to occur in SANS. The earliest manifestation of syringomyelia, before the cyst develops, is cord edema^52^ (see Fig. 3A, B). Similarly, there are alterations in water diffusivity, indicating increased free water within the white matter throughout the brain secondary to spaceflight^53^. Increasing the compliance of the spinal canal by posterior fossa decompression can eradicate both the cyst and edema in syringomyelia, suggesting the compliance change is causative (see Fig. 3C). The optic nerves have a glymphatic clearance system similar to the brain and the spinal cord. A mouse model showed CSF tracer uptake into the optic nerve via the perivascular spaces^54^ with interstitial water preferentially cleared by the perivenous spaces^55^. In patients with unilateral papilledema, abnormal protein levels are observed within the optic nerve sheath CSF pool, as compared to the global CSF pool, in both IIH and in normal-tension glaucoma, suggesting obstruction of the CSF flow between the intracranial and optic nerve subarachnoid compartments^56^. Thus, the findings in syringomyelia suggest the likely cause of the often unilateral eye findings in SANS i.e. localised isolation of the affected optic nerve sheath, reduced compliance locally and glymphatic obstruction secondary to local venous dilatation (see Fig. 1). The hypothesis of relative optic nerve sheath isolation in SANS has been put forward previously. It was suggested that CSF accumulates in the optic nerve sheath due to a one-way valve mechanism in SANS^57^. This could be correct, and possibly occurs due to the dural wall thickening/ stiffening, as already hypothesised to occur in the sagittal sinus walls in SANS. Dural thickening of the optic nerve sheath within the optic canal would obstruct the back-and-forth CSF flow isolating the system.

## Are asthenia and “space brain fog” manifestations of glymphtic obstruction as well?

In their perspectives on asthenia in astronauts and cosmonauts, NASA defined the most prevalent symptoms of this disorder to be; fatigue, sleep disturbance, somatic symptoms, difficulty concentrating, decreased occupational performance and irritability^58^. They noted, there seems to be a very high probability that at least partial asthenia will develop in astronauts after 6 or more months in space^58^. Astronauts commonly experience “space fog”, which manifests as attention lapses, short-term memory problems, confusion when performing two tasks together and psychomotor problems^59^. On the day of landing, astronauts experience a general post-flight malaise in motor function and a lack of cognitive reserve, said not to be due to the fatigue component alone. These changes recover to baseline by four days after landing^60^. Fatigue is a component of MS symptomatology. The symptom of fatigue is a significant lack of physical and/or mental energy that is perceived by the individual to interfere with their usual or desired activity. It is a subjective feeling of physical, cognitive or psychosocial exhaustion and tiredness, which can be perceived by the patients at rest^61^. Fatigue in MS has a prevalence of up to 81%^62^. Fatigue in MS is often described as “brain fog” by the patients themselves. In multiple sclerosis, fatigue severity correlates with the number, site and size of the enlarged cerebral perivascular spaces^63^. Fatigue in MS also correlates with the size of the dilated cortical veins^45^. This raises a possible glymphatic cause for the fatigue in MS and perhaps also the asthenia in astronauts. It has been suggested the glymphatic system may be involved in mediating fatigue by facilitating macromolecular toxin clearance from the sleeping brain^64^.

Sleep increases glymphatic flow, while sleep deprivation reduces glymphatic flow^65^. There is a coherent pattern of oscillating electrophysiological, hemodynamic and CSF dynamics in slow wave sleep, most likely linked to the restorative effects of sleep^66^. Hyde et al. were the first to suggest there was a link between dilated perivascular spaces in the brain and chronic fatigue syndrome^67^. More recently it has been hypothesised there is a glymphatic disruption with toxin build-up in at least some cases of chronic fatigue syndrome^68^ and post-COVID-19 syndrome^69^, both of which are associated with fatigue. There is subsequent evidence of glymphatic system dysfunction even in recovered patients with mild COVID-19^70^. It has been suggested the reason fatigued individuals, such as those with chronic fatigue syndrome, complain of non-restorative sleep is both that the refreshing part of nonrapid eye movement sleep is altered, with a decrease in parasympathetic activity^71^ and the glymphatic clearance system is impaired^72^. Indeed, in chronic fatigue syndrome/ myalgic encephalitis, there is also anecdotal evidence of venous outflow dilatation, which could impair glymphatic outflow^73^. In space missions, there is a reduction in sleep time, reduced slow-wave sleep and changes in the architecture of the sleep^74^. Thus, lack of sleep could partly explain the asthenia seen in spaceflight by reducing the sleep-induced glymphatic flow. However, it is also likely that a combination of any sleep reduction together with venous outflow dilatation could be acting in synergy to alter the glymphatic system in spaceflight compounding the fatigue.

### Reporting summary

Further information on research design is available in the Nature Research Reporting Summary linked to this article.

### Supplementary information

Reporting Summary

## Data Availability

All data generated or analysed during this study are included in this published article.
